# Human prion disease surveillance in Spain, 1993-2018: an overview

**DOI:** 10.1080/19336896.2021.1933873

**Published:** 2021-06-12

**Authors:** Jesús De Pedro-Cuesta, Javier Almazán-Isla, Laura Tejedor-Romero, María Ruiz-Tovar, Fuencisla Avellanal, Alberto Rábano, Miguel Calero, Fernando J. García López, for the Spanish CJD Surveillance Group

**Affiliations:** a Department of Neurodegeneration, Ageing and Mental Health, National Epidemiology Centre, Carlos III Health Institute, Madrid, Spain; b Consortium for Biomedical Research in Neurodegenerative Diseases (CIBERNED), Madrid, Spain; c Department of Neuropathology and Brain Tissue Bank, Alzheimer Disease Research Unit, CIEN Foundation, Queen Sofia Foundation Alzheimer Centre, Madrid, Spain; d Chronic Disease Programme (UFIEC) , Carlos III Health Institute, Majadahonda, Madrid, Spain

**Keywords:** Transmissible spongiform encephalopathies, creutzfeldt-jakob disease, surveillance, epidemiology, prion proteins, registries, spain

## Abstract

In Spain, human transmissible spongiform encephalopathies (TSEs) have been undergoing continuous surveillance for over 25 years. In 1995, the system was launched as an EU Concerted Action, with EU surveillance network procedures being incorporated from 2002 onwards. The aim of this report was to describe performance and outcomes of this surveillance system across the period 1993–2018. Neurology and public health specialists from every region reported cases to a central hub at the Carlos III Health Institute, Madrid. In all, eight accidentally transmitted cases and five definite variant Creutzfeldt-Jakob disease (vCJD) patients were reported. All vCJD cases were diagnosed between 2005 and 2008. Two of these were family/dietary-related and spatially linked to a third. Yearly incidence of sporadic CJD per million was 1.25 across the period 1998–2018, and displayed a north-south gradient with the highest incidence in La Rioja, Navarre and the Basque Country. Genetic TSEs were observed to be clustered in the Basque Country, with a 4-fold incidence over the national rate. A total of 120 (5.6%) non-TSE sporadic, conformational, rapidly progressing neurodegenerative and vascular brain disorders were reported as suspect CJD. We conclude that TSEs in Spain displayed geographically uneven, stable medium incidences for the sporadic and genetic forms, a temporal and spatial family cluster for vCJD, and decreasing numbers for dura-mater-associated forms. The vCJD surveillance, framed within the EU network, might require continuing to cover all prion disorders. There is need for further strategic surveillance research focusing on case definition of rapid-course, conformational encephalopathies and surgical risk.

## Introduction

Human transmissible spongiform encephalopathies (TSEs) are rare neurodegenerative disorders associated with the presence of pathological prion proteins (PrPs). Creutzfeldt-Jakob disease (CJD), the most frequent TSE, exists in three forms: sporadic CJD (sCJD), of unknown aetiology; genetic CJD (gCJD), i.e. caused by mutations in PrP-encoding gene, *PRNP*; and acquired CJD, either variant (vCJD) or accidentally transmitted (atCJD) [1]. The world’s first CJD surveillance system was introduced in the United Kingdom (UK) on 1 May 1990, essentially for the purpose of assessing the extent to which potential changes in CJD epidemiological characteristics were linked to the occurrence of bovine spongiform encephalopathy (BSE) [2]. In the rest of Europe, CJD surveillance started in 1990 as a European Union (EU) Concerted Action, in the form of collaboration between the UK Surveillance Unit and ad-hoc surveillance groups in several EU Member Countries [3]. After the first cases of vCJD were reported in the UK in 1996 [4], the consortium expanded to become the CJD International Surveillance Network (formerly the European Creutzfeldt-Jakob Disease Surveillance Network/EuroCJD), which encompassed European Economic Space (EES) countries. This structure, coordinated by the European Centre for Disease Prevention and Control (ECDC), is still currently active in 26 countries [5].

Over a period of almost thirty years, EuroCJD groups have done clinical research and incorporated advances in diagnosis through genetic assessment, 14-3-3 protein testing, magnetic resonance imaging (MRI), RT-QuIC testing, and risk factor identification. This has enabled EuroCJD to define diagnostic criteria on which to base both surveillance and advice on preventing transmission in medical settings. In parallel, these three decades have witnessed definite progress in diagnosis and aetiological research in the field of other conformational proteinopathies, particularly neurodegenerative, metabolic and vascular disorders [6]. Accordingly, this paper aims to describe CJD surveillance services and findings in Spain across the period 1993–2018, and propose a rationale for future work.

## Methods

### Study population and access to neurological health resources

Spain’s National Health Service, decentralized since 2002, affords universal coverage and free access to healthcare for an ageing population of around 46 million. During the surveillance period, healthcare service indicators of equity, efficiency and quality were good within the EU context [7] and the neurologist/population ratio was approximately 1/20,000.

### Early exploratory surveillance work

The preliminary findings of atCJD clusters linked to Lyodura® dura mater implants in Madrid and Murcia in the early 1990s [8] prompted the National Epidemiology Centre (NEC) at the Carlos III Health Institute to develop a CJD surveillance system and join the above-mentioned EU Concerted Action. The neuropathology group of the Spanish Neurology Society examined CJD post-mortem studies but did not find results supporting the presence of other atCJD clusters.

In 1995, the NEC requested all hospital neurology units in the country to collect summary clinical data on CJD cases with clinical onset from 1993 onwards. The stated purposes of CJD surveillance at such an early point in time were to monitor incidence of CJD, describe the clinical and epidemiological profile of CJD, detect accidentally transmitted cases and a potential relationship with BSE, and identify new risk factors. Once vCJD appeared [4], however, the Spanish CJD surveillance system went from being a research-driven enterprise run voluntarily by neurologists to becoming a legally regulated, public health activity.

### State-centred, EU-coordinated CJD surveillance in Spain

In 1996, the Spanish Government issued an order for a CJD registry to be created, using data notified by the country’s 17 regional systems [9]. The Spanish working group joined the international surveillance system, under the acronym EuroCJD. In addition to surveillance data in Spain, EuroCJD reported incidence figures for various CJD forms, diagnostic trends, molecular genetics, and analysis of health-occupational risk incidents or regional views in an EU context [10–13]. The purpose of CJD surveillance and human resources allocated during the study period remained unchanged in Spain, despite the fact that at an EU level, CJD surveillance objectives were restricted to vCJD from 2011 onwards.

#### Surveillance protocol

The reporting protocol, which has been in use since 1996, was inspired by the first case-control study on CJD risk factors [14]. National Health Service experts drew up national diagnosis and surveillance guidelines, which were published in 2003 [15]. Since 2005, all vCJD suspects have been examined using an adapted version of the expanded questionnaire administered in the UK.

#### Data flow

In general, reporting physicians, whether in public or private practice, whether alone or in collaboration with hospital epidemiologists, completed the same protocol and reported to public health co-ordinators and regional authorities. Every regional authority also nominated a clinical co-ordinator, who acted as a consultant. Regional registries reported to the National CJD Registry central hub, located at the NEC, and held annual meetings. In turn, the National CJD Registry and the Ministry of Health were jointly responsible for international coordination.

#### Diagnostic work-up and criteria

A few laboratories, validated by the EU for genetic testing and 14-3-3 protein analysis in cerebrospinal fluid (CSF) at the Carlos III Health Institute and other centres, provided support for CJD diagnosis. We used updated criteria drawn up for international CJD surveillance [16]. In Spain, we applied criteria modification in retrospect to possible sCJD patients from whom frozen CSF samples had been stored, in order to carry out 14-3-3 test analyses. The reason for this was that a few potential sCJD cases with atypical EEG (electroencephalogram) and no stored DNA were reclassified on the basis of family history and positive 14-3-3 protein as probable gCJD. Diagnostic criteria were updated in 2003, when epidemiological criteria changed (i.e. vCJD risk from exposure to blood products), and again in 2010, when MRI criteria were incorporated [17]. RT-QuIC tests were generally unavailable in Spain before 2018. In some instances, the UK unit provided support for neuropathology and RT-QuIC tests. The laboratory results were periodically updated by surveillance personnel at the National CJD Registry. A few specially prepared centres performed post-mortem studies. Altogether, nine regional laboratories performed neuropathological autopsies in suspected CJD cases from their own territory, most of them located in the north half of Spain or in the Balearic or Canary Islands. A national reference centre in Madrid received cases from all other regions. All involved laboratories were linked to human brain banks.

#### Dissemination of surveillance results

Periodically, we updated summary statistics reports on the Carlos III Health Institute website. We calculated crude and age-adjusted incidence rates for regions and the country as a whole, using annual official populations and the 1975 European standard population, and have been issuing yearly on-line evaluation reports since 2000.

#### Incidents

At a national level, a specialized group (designated as the CJD Technical Group) received data on incidents reporting high-risk situations, such as those implying potential transmission at hospitals due, mainly, to contaminated endoscopic or invasive surgical instruments. This group, co-ordinated by the surveillance unit at the Carlos III Health Institute, released several internal technical reports for specific situations flagged by clinicians and hospital preventive services or the industry.

As this work involved public health surveillance, not research, we considered it unnecessary for a research ethics committee to review it.

## Results

### Surveillance system performance at specific time intervals

#### Assessment of surveillance services

*1998–2000*. Five years after the system had been implemented, regional points were surveyed in 2001 to assess their performance, using a structured region-based questionnaire drawn up with *EuroCJD* support. Its findings were as follows:

- in terms of human resources, the average annual work time in person-months per million inhabitants over the period 1998–2000 was 0.82 (0.50, epidemiologists; 0.20, neurologists; 0.10, administrative; and 0.02, other);
- in 1998, 92% of funding came from public health services and 8% from research grants;
- it was only from 2000 onwards, when the first BSE cases were diagnosed in Spanish cattle, that notification was made compulsory;
- complementary case-finding methods were frequently used at the regional level, ranging from hospital discharge records to death records and 14-3-3 test lab data; and lastly,
- based on a perusal of laboratory records for 14-3-3 tests and ad-hoc surveys, the total proportion of CJD underreported by regional clinical and epidemiological co-ordinators was estimated to be 12% and seen to display an uneven geographical pattern.

Regional surveillance personnel systematically carried out a follow-up of reported suspects or cases, based on examination of vital statistics, contact with reporting physicians and, rarely, contact with relatives. Furthermore, surveillance team members made 153 registered home visits to patients, i.e. 3.9 per million population. As regards potential missing cases in 1998, while the number of patients with a final diagnosis of probable sCJD, without periodic sharp wave complexes or with no EEG performed, totalled 12, the estimated number of missing referrals was 3. As for cases aged under 50 years at clinical onset, we found 2 reported sCJD cases but no estimated misses for sCJD or vCJD. Post-mortems of probable and definite sCJD patients with clinical onset in 1998 were performed in 50 cases (54%)

#### Clinical work-up

From 1998 onwards, 14-3-3 tests were performed in CSF and EEG in most cases, and genetic testing was conducted in 70% of referrals [12]. For the period 1993–2002, the *PRNP* gene mutations reported were E200K and D178N, thus mimicking the patterns of other EU countries. Our genetic TSE (gTSE)/sCJD ratio was 18/380, in contrast to that registered for Slovakia (41/18). PrP typing was occasionally done [12].

#### Diagnostic pattern of non-CJD referrals

In 2014, 104 post-mortems examined non-CJD cases diagnosed during the period 1995–2013 in Spain and initially reported to the National CJD registry as suspected CJD [18]. Out of these, the neuropathological diagnosis in 76 cases was classified as follows: neurodegenerative, 14; vascular, 20; mixed (neurodegenerative + vascular), 23; and other or not classified, 19 [18].

### Surveillance outcomes

**Figure 1.**
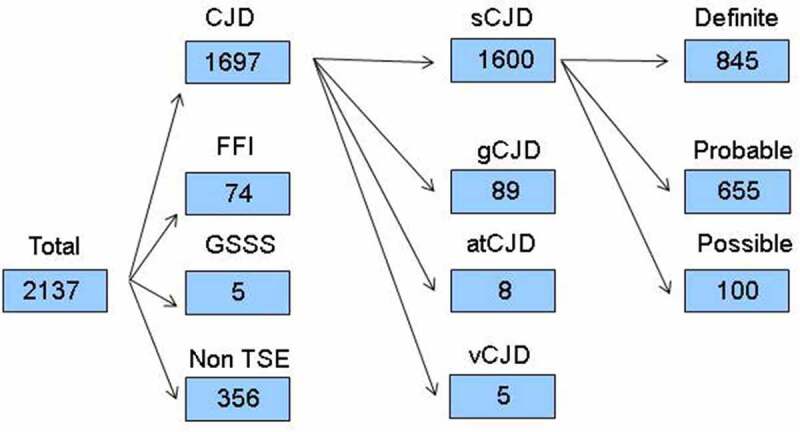
Diagram flow from suspect TSE notifications for 1993–2018. TSE indicates transmissible spongiform encephalopathy; sCJD, sporadic Creutzfeldt-Jakob disease (CJD); gCJD, genetic CJD; atCJD, accidentally transmitted CJD; vCJD, variant CJD; FFI, fatal familial insomnia; GSSS, Gerstmann-Sträussler-Scheinker syndrome

Up until 31 December 2018, of 2137 referrals registered: 356 (17%) were classified as non-TSE and 1781 (83%) as TSE cases: 1600 (75%) were classified as sCJD, of which 845 (53%) were definite, 655 (41%) probable and 100 (6%) possible; 8 were classified as atCJD (0.4%) due to dura mater; 5 (0.2%) were classified as vCJD; and 168 were classified as gTSEs, broken down into 89 (4%) gCJD, 74 (5%) fatal familial insomnia and 5 (0.2%) Gerstmann-Sträussler-Scheinker syndrome (Figure 1). The mean time interval between symptom onset and referral reception at the NEC after exclusion of non-TSE cases was 12.3 months.

**Figure 2.**
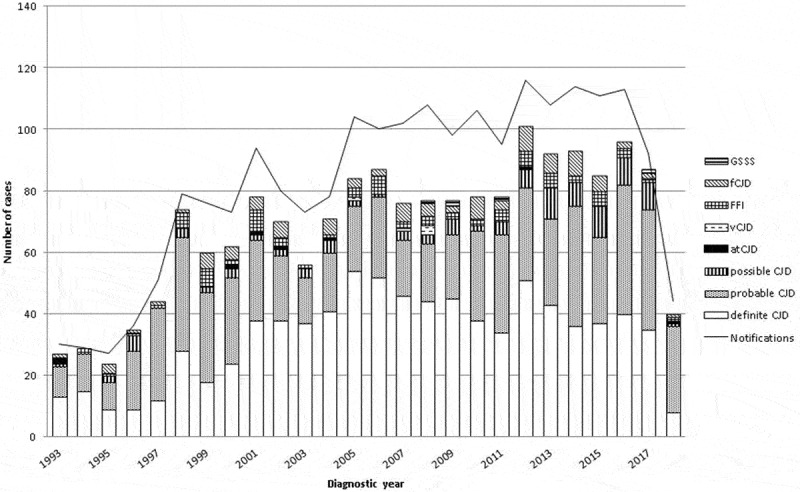
Annual number of notifications as at 31 December 2018 and different diagnostic categories by year of diagnosis. sCJD indicates sporadic Creutzfeldt-Jakob disease (CJD); gCJD, genetic CJD; atCJD, accidentally transmitted CJD; vCJD, variant CJD; FFI, fatal familial insomnia; GSSS, Gerstmann-Sträussler-Scheinker syndrome

Annual TSE notifications across the period 1993–2018, classified by TSE category, diagnostic year and certainty are depicted in Figure 2. The Table 1 shows this information with sCJD sex-specific details. Annual numbers of notifications and deaths ran relatively parallel, in that numbers were low before 1998, atCJD was observed up to 2004, and vCJD appeared in 2005 with the three latest cases dying in 2008. Definite sCJD appeared to level off after 2008. Numbers in 2010 were in general lower, particularly for post-mortem confirmed sCJD. Notification in Spain was mainly based on cases at advanced time points of the diagnostic clinical work-up (data not shown).

**Table 1.** Annual numbers, notifications and subsequently confirmed and probable Creutzfeldt-Jakob disease cases and deaths: 1993–2018 (cases in 1993 and 1994 in retrospect)

Year	1993	1994	1995	1996	1997	1998	1999	2000	2001	2002	2003	2004	2005	2006	2007	2008	2009	2010	2011	2012	2013	2014	2015	2016	2017	2018	Total
Number of reports*			33	60	37	70	55	96	101	82	79	77	105	76	102	122	84	113	96	100	121	108	126	97	102	95	2137
Cases by diagnostic year	30	29	27	36	51	79	76	73	94	80	73	78	104	100	102	108	98	106	95	116	108	114	111	113	92	44	2137
Cases by year of death	24	19	28	36	33	70	67	61	84	70	68	74	91	81	101	99	86	89	85	103	102	93	97	87	89	46	1883
Men, sCJD																											
Definite	6	7	3	5	2	15	8	10	17	18	15	25	31	25	18	17	20	16	15	25	24	17	20	15	18	3	395
Probable	4	5	3	11	12	16	9	12	12	8	4	4	7	11	7	8	9	13	15	14	14	20	13	23	15	13	282
Probable + definite	10	12	6	16	14	31	17	22	29	26	19	29	38	36	25	25	29	29	30	39	38	37	33	38	33	16	677
Possible	1		1	5		2	2	2	2	1	1	3	1	1	3	1	3	1	1	4	7	3	4	8	7	1	65
All	11	12	7	21	14	33	19	24	31	27	20	32	39	37	28	26	32	30	31	43	45	40	37	46	40	17	742
Women, sCJD																											
Definite	6	8	6	4	10	13	10	14	21	20	22	16	23	27	28	27	25	22	19	26	19	19	17	25	17	5	449
Probable	6	5	6	8	18	21	20	16	14	13	11	15	14	15	11	11	12	16	17	16	14	19	15	19	24	15	371
Probable + definite	12	13	12	12	28	34	30	30	35	33	33	31	37	42	39	38	37	38	36	42	33	38	32	44	41	20	820
Possible	1			1		1		1	2	1	1			2	2	1	3	2	3	5	6	1	2				35
All	13	13	12	13	28	35	30	31	37	34	34	31	37	44	41	39	40	40	39	47	39	39	34	44	41	20	855
VV/MM	0/0	0/2	0/1	2/1	2/10	8/14	4/17	6/13	6/29	8/23	8/26	13/29	7/37	8/40	10/24	9/17	8/29	9/19	10/17	5/18	5/21	8/24	6/18	4/22	6/21	5/11	157/483
VV/MM (ratio)	0	0	0	2	0.2	0.57	0.24	0.46	0.21	0.35	0.31	0.45	0.19	0.20	0.42	0.53	0.28	0.47	0.59	0.28	0.24	0.33	0.33	0.18	0.29	0.45	0.33
gTSE, all																											
Definite	1	2	0	1	0	4	5	3	9	5	0	4	4	5	6	4	3	5	4	5	7	5	7	4	2	0	95
Probable	0	0	4	1	2	2	6	3	2	3	1	2	2	3	2	4	3	4	4	8	4	5	3	1	2	2	73
Accidentally transmitted, All	2							1	1	1		1								1						1	8
Variant CJD													1		1	3											5
Non-TSE cases^a^	3	0	3	1	7	5	16	11	16	10	17	7	20	13	26	31	21	28	17	15	16	21	26	17	5	4	356
Post-mortem: neurodegenerative/vascular	1	0	1	0	1	1	7	6	8	3	7	2	8	6	13	13	7	13	7	2	2	1	9	1	1	1	121
Other clinically confirmed diagnoses	1	0	0	0	2	1	4	4	4	1	3	0	1	4	2	2	3	1	1	3	0	2	2	1	0	0	42

CJD indicates Creutzfeldt-Jakob disease; sCJD, sporadic Creutzfeldt-Jakob disease; gTSE, genetic transmissible spongiform encephalopathy; MM, methionine-methionine; VV, valine-valine; MV, methionine-valine; TSE, transmissible spongiform encephalopathy; ^a^The difference between total non-TSE cases and the addition of the last two rows is the number of cases, 193 in all, whose diagnosis was never registered.

The number of notifications and cases of different TSE categories, along with definite/probable ratios in sCJD and age-adjusted rates for regions in the period 1998–2018 are shown in the Table 2. The age-adjusted incidence of definite and probable sCJD for 1998–2018 was 1.25 per million person-years, ranging from 0.50 to 1.87 across the different regions, with the highest figures being registered by La Rioja, Navarre and the Basque Country. The age-adjusted incidence of genetic TSE for the same period was 0.16 per million person-years, with a range of 0.0 to 0.69 in the Basque Country, which accounted for 31 of the 74 registered cases of fatal familial insomnia. Remarkable differences among regions were observed for confirmation proportions (definite over total sCJD), with a mean percentage of 54.3%, ranging from 16.9% to 84.3% for Castilla la Mancha and La Rioja respectively.

**Table 2.** Notifications, definite and probable sCJD and gTSE cases with age-adjusted incidences per million person-years in 1998–2018 by regions

			sCJD					gTSE	
Regions	Population·10^−3^	Notifications	n	Incidence per million person-years	Definite/ probable ratio	MM (%)	VV+MV (%)	n	Incidence per million person-years
Andalusia (a)	166 600.03	298	210	0.97	69/122	61.8	38.2	15	0.07
Aragon (b)	26 765.78	64	41	1.08	22/19	63.6	36.4	3	0.11
Asturias (c)	22 152.33	34	30	0.81	19/9	57.1	42.9	1	0.04
Balearic Islands (d)	21 292.74	32	25	1.00	10/13	62.5	37.5	0	0.00
Canary Islands (e)	41 524.34	58	51	1.12	14/36	72.4	27.6	0	0.00
Cantabria (f)	11 825.76	26	23	1.41	16/7	83.3	16.7	1	0.00
Castile la Mancha (g)	40 576.72	95	65	1.16	11/46	55.2	44.8	9	0.17
Castile and León (h)	51 910.37	162	123	1.42	63/56	50.9	49.1	13	0.30
Catalonia (i)	146 771.14	325	290	1.51	209/75	62.2	37.8	32	0.19
Valencian Community (j)	97 879.48	330	219	1.43	85/97	55.2	44.8	13	0.12
Extremadura (k)	22 559.49	37	25	0.78	11/12	50.0	50.0	0	0.00
Galicia (l)	57 047.07	103	84	0.90	46/35	71.8	28.2	2	0.03
Madrid (m)	126 107.99	271	205	1.27	127/70	68.9	31.1	34	0.23
Murcia (n)	28 529.56	48	39	1.22	16/21	82.6	17.4	2	0.00
Navarre (o)	12 600.51	39	32	1.77	25/6	33.3	66.7	5	0.35
Basque Country (p)	44 589.39	195	121	1.72	90/26	50.0	50.0	38	0.69
La Rioja (q)	6 291.76	19	16	1.84	12/4	50.0	50.0	0	0.00
Ceuta (r)	1 596.38	0	0	0.00	0/0	0.0	0.0	0	0.00
Melilla (s)	1 534.93	1	1	0.50	0/1	0.0	0.0	0	0.35
TOTAL	928 155.77	2137	1600	1.25	951/730	61.1	38.9	168	0.16

sCJD indicates sporadic Creutzfeldt-Jakob; gTSE, genetic transmissible spongiform encephalopathy; MM, methionine-methionine; VV, valine-valine; MV, methionine-valine.

**Figure 3.**
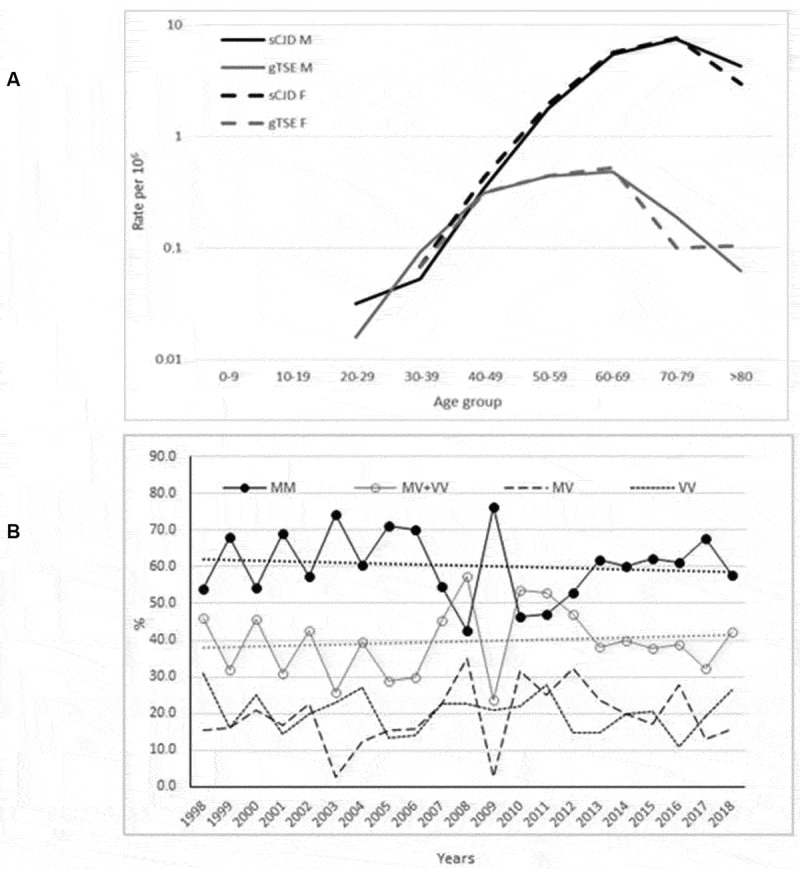
(a) Age- and sex-specific incidences of definite and probable sporadic Creutzfeldt-Jakob disease (sCJD) and genetic transmissible spongiform encephalopathy (gTSE), for 1998–2018; and (b) time trends for annual genetic subgroup proportions in percentages

The epidemiologically significant features of the comparison between gTSE and sCJD are depicted in Figure 3. Firstly, the age-specific incidences of sCJD and gTSE shown in the top part of Figure 3 for men and women across the different study periods indicate that sCJD had an older age at onset and higher incidence rates than did gTSE. Secondly, it will be seen from the bottom part of Figure 3 that time trends for *PRNP* codon 129 genotypes of confirmed and probable sCJD remained invariant. The percentages of *PRNP* codon 129 genotypes MM and others (MV+VV) were 61.1% and 38.9%, respectively. No regional patterns were in evidence.

vCJD was first confirmed in 2005 in a 26-year-old woman in Madrid. Four other cases were diagnosed in 2007–2008, three in the province of León and one in Cantabria. Among the cases in León, a mother and son aged 64 and 40 years at onset, respectively, were diagnosed within a short interval [19]. They shared dietary habits, including bovine brain and offal, dating back decades, and had no significant stay in the UK or recorded history of blood transfusion, grafts or surgery. Two other suspect cases of TSE in young persons with a residential history or current residence in León were diagnosed with definite sCJD or possible sCJD without a post-mortem study; both were MM homozygous at *PRNP* codon 129 and had a disease duration longer than that of the sporadic forms.

**Figure 4.**
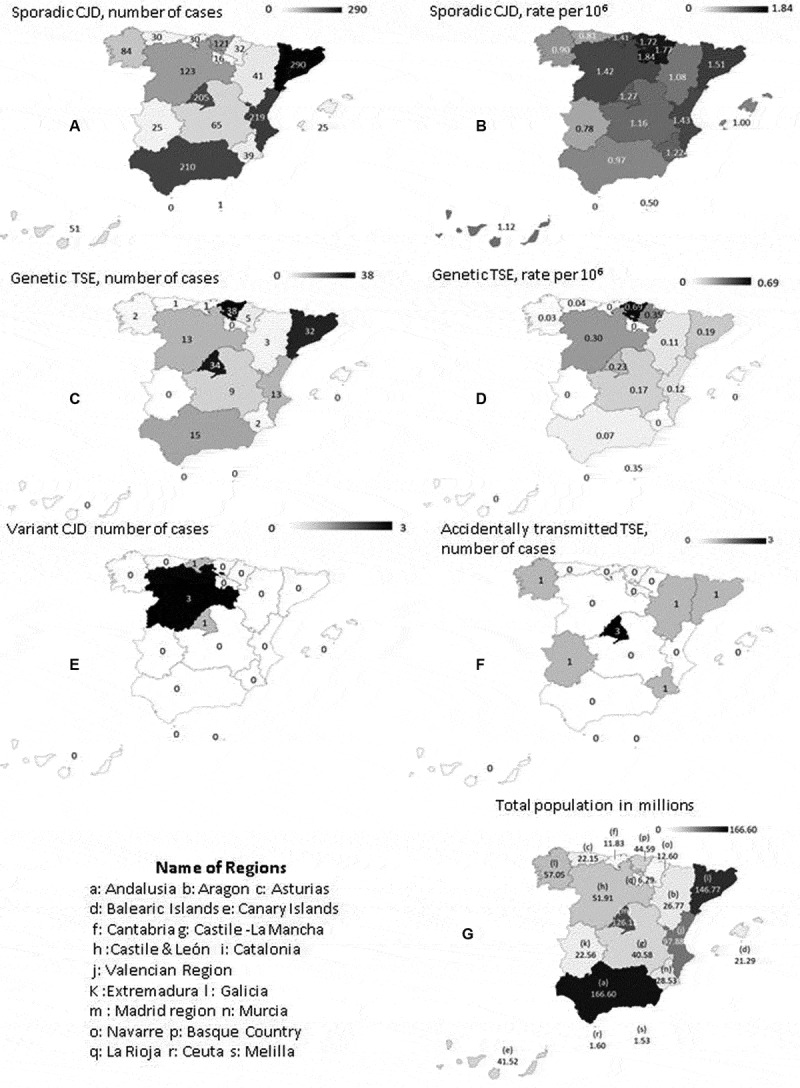
Geographical patterns in Spain of: (a) sporadic Creutzfeldt-Jakob disease (sCJD), number of cases (b) sCJD age-adjusted incidence; (c) genetic transmissible spongiform encephalopathy (gTSE), number of cases; (d) gTSE incidence; (e) variant Creutzfeldt-Jakob disease (vCJD), number of cases; (f) accidentally transmitted Creutzfeldt-Jakob disease (atCJD); and (g) population in millions

Geographical patterns of sCJD cases, sCJD age-adjusted incidence, gTSE cases, gTSE age-adjusted incidence, vCJD cases, atCJD cases, and regional populations are depicted in Figure 4. The highest 1998–2017 incidences of both sCJD and gTSE were seen in the north central regions, Cantabria and the Basque Country. The lowest sCJD incidence rates were seen in the south, particularly in Andalusia and Castile-La Mancha. Mutations at *PRNP* gene were described in 147 patients, in whom the most frequent mutations were D178N (n = 74) and E200K (n = 65). We also investigated an sCJD cluster in Las Palmas, Grand Canary Island, with inconclusive causal findings.

By year-end 2018, a total of 356 CJD suspects had eventually been diagnosed as non-TSE cases (Table 1). Of these, 121 had neuropathology studies, with all being classified as degenerative or mixed vascular-degenerative dementia; 42 had alternative clinically confirmed diagnoses; and 193 had uncertain diagnosis. A breakdown by type of deposit showed that tauopathies, beta-amyloidosis, and alpha-synucleinopathies were dominant.

## Discussion

Since 2005, CJD surveillance, integrated with ECDC harmonized surveillance networks encompassing 21 countries, has compiled a comparatively large number of clinical variables [20]. After three decades of surveillance of an initially non-existent, potentially emerging disorder (vCJD), its scope exceeds the public health services original framework, and represents a unique platform for the future, given the formidable current expansion of knowledge on conformational disorders.

*Sporadic CJD*. sCJD was the only entity with an annual incidence high enough to be accurately monitored, with sCJD incidence in Spain ranking medium among EU countries. The increases in 1996, 1998 and 2001 could possibly have been determined by better surveillance due, respectively, to: (a) the first report of the vCJD epidemic in the UK in 1996; (b) the widespread use of CSF diagnostic tests in 1998; and (c) the report of the first Spanish BSE cases in November 2000. The low count in 2018 could be explained by notification and post-mortem delays. The clinical profile of sCJD in Spain fits well with that of sCJD in EU countries, in terms of both clinical presentation and median survival, 5.0 months [21]. However, sCJD survival in Spain differed from that described for sCJD in Japan [22] and Taiwan [23], where patients with sCJD had around a threefold survival time. In Spain, an extremely atypical definite case with onset at 11 years of age remains aetiologically unexplained. Healthcare occupations in Spain did not appear to pose a higher risk of sCJD [13]. Incident investigations did not reveal clues for sCJD transmission.

*AtCJD*. The incidence of atCJD linked to dura mater grafts, either disseminated or clustered in Murcia, vanished after 2004. The cluster in Murcia, contrary to what was observed in Japan, did not include implants manufactured before 1978 and not withdrawn from the Spanish market, which constituted a preliminarily working hypothesis in 1992. A case of this cluster not included in the initial report [8] was captured by surveillance in 2002.

*Genetic TSE*. Incidence of gTSE was remarkably high in the Basque Country, corresponding mainly to the D178N mutation, which is also relatively common in Spain and in European countries [24]. A proposed surgical link between the high prevalence of mutations in the Basque Country and the high sCJD incidence was examined by local epidemiologists with inconclusive results.

*Variant CJD and its relationship with BSE*. The five vCJD cases reported in Spain, a scant number for efficient statistical testing, constituted a unique sample clustered by space and family aggregation, and heterogeneous by age at onset, with a comparatively late peak in 2008 when compared to other countries [19]. The link with BSE suggested by infection in mouse models [25] was consistent with imports of beef from the UK until 1996, indirect evidence of import of BSE-tainted beef and dietary exposure linked to occupation of the first case in Madrid, and lastly, by dietary exposure to bovine brain by a mother and son with residence in a region where BSE was frequent and animal slaughter took place at a domestic level. Two cases occurred in young residents of the adjacent Portuguese area where BSE was also frequent. It could be speculated that the Spanish vCJD cases in 2005–2008 might have corresponded to two types of exposure to BSE tissues, the first linked to imported BSE cattle from the UK [26], and the second to two cases exposed to local BSE in León province, possibly secondary to the import of bone meal from the UK after 1985 [26]. The time- and space-clustered onsets in 2007–2008 and the lack of other suspect vCJD from 2008 onwards point to a restricted outbreak in León province.

*Non-TSE cases*. The 16.3% of notifications during the period 1993–2018 which resulted in the exclusion of TSE diagnoses was considerably low, when compared to systems in Germany or the UK, which reached approximately 50% of counts, or in France with an even higher proportion. The majority of our non-TSE cases were conformational proteinopathies which corresponded to a modest proportion of rapid-course dementias captured by surveillance, due to clinical similarities with CJD [27]. Delays in reporting CJD suspected cases might go to explain such a low figure.

*New risk factors*. The Spanish group participated in EuroCJD collaborative research projects relating to clinical, genetic, and risk (health-related occupations, invasive procedures) factors [10,28], and drew up the relevant technical reports. The potential risks from surgery and blood products warrant the implementation of preventive measures in medical settings [29,30].

*The future of TSE surveillance*. As suggested from differences in time trends in *PRNP* 129 codon genotypes as compared to Germany [31], TSE surveillance in Spain can be improved in ascertainment of sCJD subtypes and in reporting from low sCJD incidence regions. Budka and Will have provided powerful reasons for continuing TSE surveillance within the EU network, namely: animal sources for human prion infection other than BSE cannot be excluded; the potentially increasing circulation of prions between humans by blood, blood products and medical procedures; the prevalence of vCJD prion carriers in the UK; and lastly, the scientific study of prion diseases as a paradigm for other neurodegenerative disorders with ‘prion-like’ spread of pathological proteins [32]. Several factors may represent a risk in Spain since exposure to BSE in the diet was relevant, blood-donation restrictions in León were not taken, occupational risk for vCJD in laboratory workers cannot be ruled out, and there may be excess risk for haemophiliacs in Central Spain due to the well-established pharmacological exposure of a few hundred people. The presence of chronic wasting disease in wild cervids is uncertain. The unique features of vCJD in Spain, such as the late age at onset, supports the notion of surveillance of all TSE entities as a requirement for proper clinical identification of vCJD cases.

**Figure 5.**
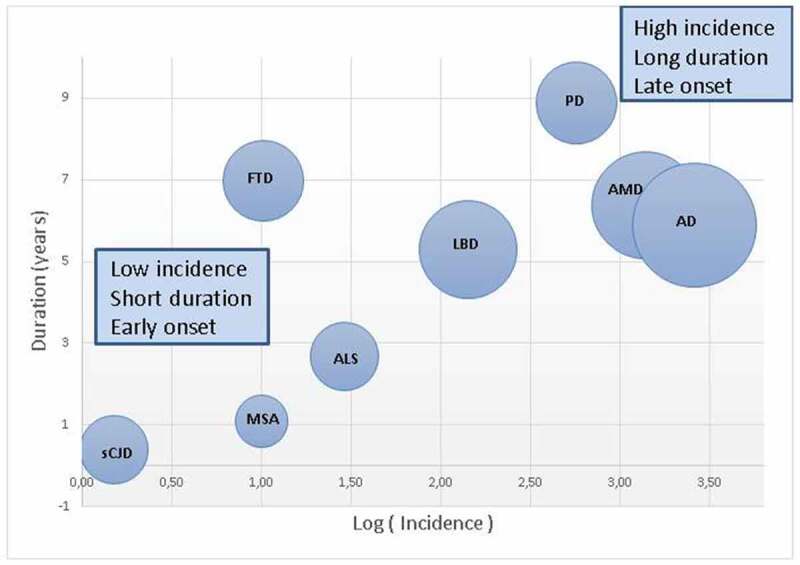
Relationship as between incidence, disease duration and onset. The diameter of each bubble is computed as the mean age at onset minus 60 years. Shown in the top right corner are disorders with high incidence, long duration and later onset (AD, AMD, PD and LBD). Shown at the bottom left are disorders that are more rapidly progressive, with earlier onset and lower incidence (sCJD, MSA and ALS). sCJD indicates sporadic Creutzfeldt-Jakob disease; MSA, multisystemic atrophy; FTD, frontotemporal dementia; ALS, amyotrophic lateral sclerosis; LBD, Lewy body dementia; PD, Parkinson´s disease; AMD, age-related macular degeneration; AD, Alzheimer’s disease. Data from figures cited in ref [33]

The epidemiology of neurodegenerative disorders (NDD) and dementia, both general and clinical, was initially built on a syndromic basis and subsequently on fragmented clinical, neuropathological and molecular views. CJD surveillance work in large EU Member States has revealed the presence of rapid-course, sporadic NDD/dementia which has never been epidemiologically studied and might share transmission mechanisms with prion disorders [33]. Strategically oriented EU research focusing on NDD subgroups relevant for public health, particularly those identified or not in Figure 5, with low incidence, short survival and earlier onset, may constitute an avenue for building a rationale of CJD in-parallel surveillance in the future.

To sum up, TSE surveillance in Spain indicated geographically uneven, stable, medium-level incidences for the sporadic and genetic forms, revealing a small spatial/temporal cluster of variant CJD before 2009, decreasing numbers of dura-mater-associated forms, and a considerable, spatially widespread, number of conformational, rapidly progressing neurodegenerative and vascular disorders. Room for surveillance improvement exists both in specific regions and, conditional on strategic research in rapid-course dementia/NDD, also at an EU level.

## Statements

The Spanish CJD Study and Surveillance Group (Grupo para el Estudio y Vigilancia de las EETH del Consejo Interterritorial del Sistema Nacional de Salud) is composed of:

— Spanish National Register of Human Transmissible Spongiform Encephalopathies (Almazán J, Avellanal F, Calero M, De Pedro-Cuesta J, Martínez P, Ruiz M, Tello O, García López FJ.), National Epidemiology Centre and Unit of Spongiform Encephalopathies, National Microbiology Centre, Health Institute Carlos III, Madrid.

— Department of Pathology, University Hospital Fundación Alcorcón (Guerrero C, Rebolledo A), Alcorcón.

— CIEN Foundation, Foundation Research Centre of Neurological Diseases (Calero M, Rábano A).

— Regional epidemiologic and clinical coordinators in 2020: *Andalusia*: Heras JA, Varo A, Guillén J; *Aragon*: Alonso JP, Juanas FE, Ladrero O, López del Val J; *Asturias*: Huerta, I, Margolles M, Asensi A; *Balearic Islands*: Grau P, Nicolau A, Sureda B; *Canary Islands*: Izquierdo A, C. Muñoz, Pérez MC; *Cantabria*: Galán M, Martínez F, Polo JM; *Castile-La Mancha*: Cabeza C, Gutiérrez G, Humanes S; *Castile and León*: Goñi M, Marcos H; *Catalonia*: Domínguez A, Nos C; *Valencian Region*: Escudero J, Guiral S, Marín C; *Extremadura*: Casado I, Fuentes A, López M. Ramírez JM, Sánchez JF; *Galicia*: Losada I, Malvar A, Navarro C, Robles A; *Madrid*: Del Ser T, Frank A, Muñoz J, Pichiule M, Ramírez R; *Murcia*: Barranco MI, Castán Y, Contreras MA, García-Fulgueiras A, Pina R; *Navarre*: Barricarte A, Castilla J, Tuñón I; *Basque Country*: Arteagoitia JM, Zarranz JJ; *La Rioja*: Blanco A, Gil A, Martínez E, Marzo ME, Perucha M; *Ceuta*: Carrillo FJ, Rivas AI; *Melilla*: Castrillejo D, Duque G.

## References

[1] Prusiner SB. The prion diseases. Brain Pathol. 1998;8(3):499–513. PMID: 9669700. 9669700 10.1111/j.1750-3639.1998.tb00171.x PMC8098303

[2] The UK National Archives. The BSE inquiry: the report. National Archives UK BSE inquiry. 25 5 2006. Available from: https://webarchive.nationalarchives.gov.uk/20060525120000/http://www.bseinquiry.gov.uk/report/volume8/toc.htm. Accessed on 2021 216.

[3] Alperovitch A, Brown P, Weber T, et al. Incidence of Creutzfeldt-Jakob disease in Europe in 1993. Lancet. 1994;343(8902):918. PMID: 7908378. 10.1016/s0140-6736(94)90037-x 7908378

[4] Will RG, Ironside JW, Zeidler M, et al. A new variant of Creutzfeldt-Jakob disease in the UK. Lancet. 1996;347(9006):921–925. PMID: 8598754. 8598754 10.1016/s0140-6736(96)91412-9

[5] European Centre for Disease Prevention and Control. European Creutzfeldt-Jakob Disease Surveillance Network (EuroCJD). European Centre for Disease Prevention and Control, 2021. Available from https://www.ecdc.europa.eu/en/about-us/who-we-work/disease-and-laboratory-networks/european-creutzfeldt-jakob-disease Accessed on 2021 216.

[6] Westermark GT, Westermark P. Prion-like aggregates: infectious agents in human disease. Trends Mol Med. 2010;16(11):501–507. PMID: 20870462. 20870462 10.1016/j.molmed.2010.08.004

[7] García-Armesto S, Abadía-Taira MB, Durán A, et al. Spain: health system review. Health Syst Transit. 2010;12(4):1–xx. PMID: 21224176. 21224176

[8] Martínez-Lage JF, Poza M, Sola J, et al. Accidental transmission of Creutzfeldt-Jakob disease by dural cadaveric grafts. J Neurol Neurosurg Psychiatry. 1994;57(9):1091–1094. PMID: 8089676. 8089676 10.1136/jnnp.57.9.1091 PMC1073134

[9] Spain’s Ministry of Health and Consumption. Orden de 21 de octubre de 1996 por la que se amplía la de 21 de julio de 1994 por la que se regulan los ficheros con datos de carácter personal gestionados por el Ministerio de Sanidad y Consumo (2016). Available from https://www.boe.es/eli/es/o/1996/10/21/(1). Accessed on 2021 216.

[10] Saiz A, Nos C, Yagüe J, et al. The impact of the introduction of the 14-3-3 protein assay in the surveillance of sporadic Creutzfeldt-Jakob disease in Catalonia. J Neurol. 2001;248(7):592–594. PMID: 11518001. 11518001 10.1007/s004150170137

[11] Kovács GG, Puopolo M, Ladogana A, et al. Genetic prion disease: the EUROCJD experience. Hum Genet. 2005;118(2):166–174. PMID: 16187142. 16187142 10.1007/s00439-005-0020-1

[12] De Pedro-Cuesta J, Glatzel M, Almazã¡n J, et al. Human transmissible spongiform encephalopathies in eleven countries: diagnostic pattern across time, 1993-2002. BMC Public Health. 2006;6(1):278. PMID: 17096829. 17096829 10.1186/1471-2458-6-278 PMC1665456

[13] Alcalde-Cabero E, Almazan-Isla J, Brandel JP, et al. Health professions and risk of sporadic Creutzfeldt-Jakob disease, 1965 to 2010. Euro Surveill. 2012;17(15). 10.2807/ese.17.15.20144-en PMID: 22516047. 22516047

[14] Van Duijn CM, Delasnerie-Laupretre N, Masullo C, et al. Case-control study of risk factors of Creutzfeldt-Jakob disease in Europe during 1993-95. European Union (EU) collaborative study group of Creutzfeldt-Jakob disease (CJD). Lancet. 1998;351(9109):1081–1085. PMID: 9660576. 9660576 10.1016/s0140-6736(97)09468-3

[15] Spain’s Ministry of Health and Consumption. Guía ECJ. 2003. Available from: https://www.mscbs.gob.es/ciudadanos/enfLesiones/enfTransmisibles/docs/GuiaECJ.pdf. Accessed on 2021 216.

[16] Zerr I, Pocchiari M, Collins S, et al. Analysis of EEG and CSF 14-3-3 proteins as aids to the diagnosis of Creutzfeldt-Jakob disease. Neurology. 2000;55(6):811–815. PMID: 10994001. 10994001 10.1212/wnl.55.6.811

[17] Zerr I, Kallenberg K, Summers DM, et al. Updated clinical diagnostic criteria for sporadic Creutzfeldt-Jakob disease. Brain. 2009;132(Pt10):2659–2668. PMID: 19773352. 19773352 10.1093/brain/awp191 PMC2759336

[18] De Pedro-cuesta J, Rábano A, Martínez-Martín P, et al. Comparative incidence of conformational, neurodegenerative disorders. PLoS One. 2015;10(9):e0137342. PMID: 26335347. 26335347 10.1371/journal.pone.0137342 PMC4559310

[19] Riverol M, Palma JA, Alañá M, et al. Variant Creutzfeldt-Jakob disease occurring in mother and son. J Neurol Neurosurg Psychiatry. 2012;83(2):235–236. PMID: 21257982. 21257982 10.1136/jnnp.2010.232074

[20] Lenglet A, Hernandez Pezzi G. Comparison of the European Union disease surveillance networks’ websites. Euro Surveill. 2006;11(5):119–122. PMID: 16757848. 16757848

[21] Pocchiari M, Puopolo M, Croes EA, et al. Predictors of survival in sporadic Creutzfeldt-Jakob disease and other human transmissible spongiform encephalopathies. Brain. 2004;127(Pt10):2348–2359. PMID: 15361416. 15361416 10.1093/brain/awh249

[22] Nagoshi K, Sadakane A, Nakamura Y, et al. Duration of prion disease is longer in Japan than in other countries. J Epidemiol. 2011;21(4):255–262. PMID: 21628843. 21628843 10.2188/jea.JE20100085 PMC3899417

[23] Sun Y, Liu CC, Fan LY, et al. Incidence of and mortality due to human prion diseases in Taiwan: a prospective 20-year nationwide surveillance study from 1998 to 2017. Clin Epidemiol. 2020;12:1073–1081. PMID: 33116901. 33116901 10.2147/CLEP.S274093 PMC7569055

[24] Rodríguez-Martínez AB, Barreau C, Coupry I, et al. Ancestral origins of the prion protein gene D178N mutation in the Basque Country. Hum Genet. 2005;117(1):61–69. PMID: 15806397. 15806397 10.1007/s00439-005-1277-0

[25] Diack AB, Boyle A, Ritchie D, et al. Similarities of variant Creutzfeldt-Jakob disease strain in mother and son in Spain to UK reference case. Emerg Infect Dis. 2017;23(9):1593–1596. PMID: 28820380. 28820380 10.3201/eid2309.170159 PMC5572887

[26] Sanchez-Juan P, Cousens SN, Will RG, et al. Source of variant Creutzfeldt-Jakob disease outside United Kingdom. Emerg Infect Dis. 2007;13(8):1166–1169. PMID: 17953086. 17953086 10.3201/eid1308.070178 PMC2828088

[27] Geschwind MD, Shu H, Haman A, et al. Rapidly progressive dementia. Ann Neurol. 2008;64(1):97–108. PMID: 18668637. 18668637 10.1002/ana.21430 PMC2647859

[28] De Pedro-Cuesta J, Mahillo-Fernández I, Rábano A, et al. Nosocomial transmission of sporadic Creutzfeldt-Jakob disease: results from a risk-based assessment of surgical interventions. J Neurol Neurosurg Psychiatry. 2011;82(2):204–212. PMID: 20547628. 20547628 10.1136/jnnp.2009.188425 PMC3022351

[29] ECDC interim risk assessment. Questions on variant Creutzfeldt–Jakob disease and blood transfusion. 3 2010, updated 7 2011. Stockholm: ECDC, 2011. Available from https://www.ecdc.europa.eu/sites/default/files/media/en/publications/Publications/110921_TER_Risk%20assessment_vCJD.pdf. Accessed on 2021 216.

[30] European Centre for Disease Control and Prevention. Review of guidelines for prevention of Creutzfeldt–Jakob disease transmission in medical settings in EU Member States and Norway. Stockholm: ECDC, 2011. Available from https://www.ecdc.europa.eu/sites/portal/files/media/en/publications/Publications/1106_TER_Review_of_guidelines_for_prevention_of%20CJD.pdf. Accessed on 2021 216.

[31] Heinemann U, Krasnianski A, Meer B, et al. Creutzfeldt-Jakob disease in Germany: a prospective 12-year surveillance. Brain. 2007;130(Pt5):1350–1359. PMID: 17472986. 17472986 10.1093/brain/awm063

[32] Budka H, Will RG. The end of the BSE saga: do we still need surveillance for human prion diseases? Swiss Med Wkly. 2015;145:w14212. PMID: 26715203. 26715203 10.4414/smw.2015.14212

[33] De Pedro-Cuesta J, Martínez-Martín P, Rábano A, et al. Etiologic framework for the study of neurodegenerative disorders as well as vascular and metabolic comorbidities on the grounds of shared epidemiologic and biologic features. Front Aging Neurosci. 2016;8:138. PMID: 27378910. 27378910 10.3389/fnagi.2016.00138 PMC4904010

